# Supplementary material for: Fake drugs: Using Baseline Spectral Fingerprinting and a sorting algorithm to infer quality of medications
Source: PLoS One. 2023 Apr 26;18(4):e0281416. doi: 10.1371/journal.pone.0281416 (PMC10132596; doi:10.1371/journal.pone.0281416)
Supplement: Supplementary file 1 [file pone.0281416.s001.docx]

$c_{j}(a,b_{h})= \left\{\begin{matrix} 0→if g_{j}(b_{h})- g_{j}(a)≥p_{j}(b_{h}) \\ 1→if g_{j}(b_{h})- g_{j}(a)≤q_{j}(b_{h}) \\ \frac{p_{j}(b_{h})+g_{j}(a)-g_{j}(b_{h})}{p_{j}(b_{h})- q_{j}(b_{h})} Otherwise \end{matrix}\right.$

$c(a,b_{h})=\frac{\sum_{j∈F}{k_{j}c_{j}(a,b_{h})}}{\sum_{j∈F}{k_{j}}}$

$d_{j}(a,b_{h})= \left\{\begin{matrix} 0→if g_{j}(b_{h})- g_{j}(a)≤p_{j}(b_{h}) \\ 1→if g_{j}(b_{h})- g_{j}(a)>v_{j}(b_{h}) \\ \frac{g_{j}(b_{h})-g_{j}(a)-p_{j}(b_{h})}{v_{j}(b_{h})- p_{j}(b_{h})} Otherwise \end{matrix}\right.$

$σ(a,b_{h})=c(a,b_{h})\prod_{j∈F}{\frac{1-d_{j}(a,b_{h})}{1-c(a,b_{h})}}$   

  $where{: d}_{j}(a,b_{h})≥ c(a,b_{h}), 1 otherwise$   

$c_{j}(b_{h},a)= \left\{\begin{matrix} 0→if g_{j}(a)- g_{j}(b_{h})≥p_{j}(b_{h}) \\ 1→if g_{j}(a)- g_{j}(b_{h})≤q_{j}(b_{h}) \\ \frac{p_{j}(b_{h})+g_{j}(b_{h})- g_{j}(a)}{p_{j}(b_{h})- q_{j}(b_{h})} Otherwise \end{matrix}\right.$

$c(b_{h},a)=\frac{\sum_{j∈F}{k_{j}c_{j}(a,b_{h})}}{\sum_{j∈F}{k_{j}}}$

$d_{j}(b_{h},a)= \left\{\begin{matrix} 0→if g_{j}(a)- g_{j}(b_{h})≤p_{j}(b_{h}) \\ 1→if g_{j}(a)- g_{j}(b_{h})>v_{j}(b_{h}) \\ \frac{g_{j}(a)-g_{j}(b_{h})-p_{j}(b_{h})}{v_{j}(b_{h})- p_{j}(b_{h})} Otherwise \end{matrix}\right.$

$σ(b_{h},a)=c(b_{h},a)\prod_{j∈F}{\frac{1-d_{j}(b_{h},a)}{1-c(b_{h},a)}}$

  $where{: d}_{j}(b_{h},a)≥ c(b_{h},a), 1 otherwise$
