# Supplementary material for: Fake drugs: Using Baseline Spectral Fingerprinting and a sorting algorithm to infer quality of medications
Source: PLoS One. 2023 Apr 26;18(4):e0281416. doi: 10.1371/journal.pone.0281416 (PMC10132596; doi:10.1371/journal.pone.0281416)
Supplement: Attachment — Submitted filename: Response to Reviewers 5.21.20.pdf [file pone.0281416.s002.pdf]

May 21, 2020

PLOS One
Academic Editor
Levi Plaza
Suite 225
San Francisco, 94111

Response to Editorial Comments

As requested, we have updated the manuscript to include the lot number for the Praziquantel used in the study. The lot number can be found in the methods section.

Thank you,
Margaret Salmon, MD MPH
