# Supplementary material for: Fake drugs: Using Baseline Spectral Fingerprinting and a sorting algorithm to infer quality of medications
Source: PLoS One. 2023 Apr 26;18(4):e0281416. doi: 10.1371/journal.pone.0281416 (PMC10132596; doi:10.1371/journal.pone.0281416)
Supplement: Attachment — Submitted filename: Response to Reviewers PLOS 0405.docx [file pone.0281416.s003.docx]

**RESPONSE TO THE REVIEWERS**

**FROM. THE EDITOR**

1. **Formatting. - all areas of the manuscript have been checked and they meet the PLOS ONE style requirements**
2. **Financial Disclosure - completed**

5. **Review Comments to the Author**

Please use the space provided to explain your answers to the questions above. You may also include additional comments for the author, including concerns about dual publication, research ethics, or publication ethics. (Please upload your review as an attachment if it exceeds 20,000 characters)

**Reviewer #1: Very relevant use of MCDA approach for a societal problem. However, the introduction of the problem has to more clearly done, by giving the required definitions for understanding the ideas. It will be also the case for the choice of ELECTRE-Tri and the highlighting of the used methodology. References should be enriched. Figures and overall schemes would be appreciated for better getting the point of this approach. Please take time also for talking about the MCDA philosophy and its potential application in this sector**

*After the Introduction, we have added a section called “Baseline Spectral Fingerprint -	Sorting” in which we introduce the ideas of both Baseline Spectral Fingerprint and the Multi criteria sorting system. We worked to address the philosophy and applications as requested by the reviewer While we did address the ELECTRE TRI method and identified it as the ELECTRE TRI-C, we do hesitate to increase this section disproportionately at this time. This manuscript was intended for a broad group within the sectors of global health, public health, pharmaceutics and manufacturing as well as engineering.*

*Line 189 - Line 340*

**Reviewer #2: Review on PONE-D-20-14126**

**This paper provides an interesting application of multiple criteria decision aiding (MCDA) model used to infer the authenticity of medications. From a methodological point of view, this is an interesting and well-founded study. The use of ELECTRE and MCDA as a supporting methodological concept is appropriate and well explained. However, several serious concerns need to be addressed. They are mostly related to the structure of the manuscript, precision of the message, and clearness of the whole method presented in the study.**

*We agree with the reviewer and the manuscript has been restructured.*

**MAJOR REMARKS**

**1. The structure of the paper is troublesome. In the section “Methods” where you introduce us to your model and the idea behind two components, you are heavily relying on the results of the study we are yet to be introduced with. This paper needs to be significantly restructured. Either you introduce us with the methodology in a formal way and then start with your results, or you create some other smart way. This structure is not a valid one for a reader to fully understand both the method and the idea of your proposal and corresponding results.**

*The. Manuscript has been restructured to better introduce the subject and allow the reader an introduction to the concept then read about its application with Praziquantel. The “Methods” has been replaced with “Proof of Concept.” Please see below. Line 342*

*Introduction*

*Background*

*A new novel testing method*

*The Concept - Baseline Spectral Fingerprint- Sorting*

*Baseline Spectrum Fingerprint*

*MultiCriteria Sorting Algorithym*

*Proof of Concept - Praziquantel*

*Proof of Concept*

*Validation of concept*

*Discussion*

*Conclusion*

*References*

*Supplementary Materials*

**2. There are several methods within the ELECTRE family of methods. Claiming that you use ELECTRE TRI is not clear enough. The first one, at the start, was called just ELECTRE TRI (Yu, 1992). Later on, after the development of other methods, it was named ELECTRE Tri-B. Its characteristic is that the categories in the method are defined via boundary profiles set by decision-maker (DM). The newest method within the family is ELECTRE Tri-nB (Fernández et al., 2017). The “n” in the name suggests that DM is now allowed to set any number of boundary profiles between two categories. On the other hand, ELECTRE Tri-C (Almeida-Dias et al., 2010) introduced a novel way of thinking by setting, not boundary profiles, but characteristic profile, factoring as the most representative alternative for each category. In a similar fashion ELECTRE Tri-nC is a method where you can have any number of characteristic profiles.**

**Reading the paper, it becomes clear which model you use, but it is necessary to be precise. Moreover, the reason for my comment is the complete omission of the references at the moment when you introduce the method. More on reference later. Also, as ELECTRE Tri-B is a doctoral dissertation written in French, using reference (12) at that point is justified**

*The method, we chose to use is the. ELECtRE-TRI C. This is now clearly identified and referenced.*

*Line 239 - 250*

**3. I have a major problem with the organization and the names of the subsection in the “Methods” section. Are definitions necessary in the manner you introduced them? It is very uncommon, and it goes in line with a very strange structure of the paper. Moreover, you put heading as if it is the section of the same level. It is not needed as a subsection. Introduce them in brackets after the first mention like you already at that point did with the LQ/C, for example. No need to repeat yourselves.**

*We have restructured the manuscript as described above. We have removed the repetitive sections and also removed the definitions. Definitions had originally been included as this manuscript its intentionally not solely for engineers but rather a broader audience. Definitions are quite common in the literature of global health and International Non Governmental Organizations. We agree, “Definitions” may be distracting at this time.*

A**lso, naming the next subsection “Introduction to Developing Components 1,2 and Validation” without explanation of the concept is not advised, to say at least (apart from the brief mention in the introduction, but here you have to be much more precise).**

*This subsection has been retitled. Line 225 is the start*

**4. Line 131. Validation of the BSF-S method required 50 samples… Why did it “require” 50 samples precisely?**

*The sample size was not validated and was chosen by the pharmacist. We have removed any descriptors that would designate it as "required" or “calculated”. Line 464*

**5. The references in the paper are even strange and some moments. In some parts, there is a clear void where it should be supported with apparent references. The most striking example is with the notion of ELECTRE TRI, as I already mentioned. Also, when it comes to the elaboration of the ELECTRE and its (completely justified) use in your research, it should be supported with the references of applications based on the ELECTRE family of methods. Preferably in similar studies in the field, but in general as well. You can always check on most prominent journals, check the last two years of publications in top-rated journals in the OR/MS field, and you will find numerous applications of MCDA models in sorting.**

**If you find a significant number of applications in the field, it speaks in favor of your concept.**

If you do not find many applications in your field (either in medical or OR journals), it would be beneficial to elaborate on such a finding. Why is that the case? Was there a reason to opt for some other methods? Is that a gap that could be beneficial? Could this yield several novel applications in the field of the known problems? As you see, this can be a very valid point for the deeper elaboration of your concept and contribution.

*References have been added. Line 245*

6. Even though you provide “conceptual” illustrations, proper definitions would be needed. And again, in figures, you use concepts from the study and your results. Even though I understand your desire to explain the model as clearly as possible with these conceptual illustrations, I had big trouble in understanding some parts (Figure 4 as a clear favorite).

A side note is that both pictures and figures are not of good quality, you should try to make it with higher quality standards.

7. Line 205. You name subsection “Part 1 – CAT-A”. Part 1 of what? These are very uncommon terms, let alone to be used as subsections. This goes in line with comments 1 and 3.

*The subtitles you refer to have all been removed*

8. References in the paper are not according to the submission guidelines and, at some moments, not accurate. A good example is a reference (12) I already mentioned. First of all, the author is not the only editor of that handbook. Stating the date when you checked the book is not common, and it is not according to instructions. Authors need to follow guidelines to the very end and be very precise. This is not a minor mistake at all.

*The. references. have been rechecked*

9. I was very interested in the results presented in tables 2 and 3. I’ll state this example to explain my interest here. Was (Would) the conclusion of the decision-maker (and make clearer how did you derive table 3), for a wavelength 240, that there exists a difference in samples 17 and 18 with the results 0.423 and 0.414?

*Specific to the comment related to 240 nm for samples 17 and 18: the factual	substances are Splenda and Sugar, which are chemically similar, and in fact	Splenda is designed as a substitute for sugar, and this is demonstrated in the	similar readings of absorbance at not just 240, but also 250, 260 and 270 nm: For	Sugar [0.423, 0.294, 0.152, 0.129], and for Splenda [0.414, 0.288, 0.143, 0.120].*

*Specific to the Samples 17 &. 18m These two samples, as well as others in Table	2, were selected by a third party pharmacist for the purposes of calibrating the	sorting algorithm parameters, which are presented in Table 3, and I believe are	relevant to the second part of the reviewer's question: The values in Table 3 were	derived by calibrating the parameter values of the algorithm (indifference,	preference, veto and credibility threshold) towards minimizing the number of	false positives and false negatives.  In order to ensure that the parameters were	not overly broad by including chemical substances that only wildly dissimilar	absorbance profiles than Praziquantel, proxy low quality samples were included	(note Samples 2 and 11) in order to narrow the width of the absorbance pathway	to authentic Praziquantel.  The calibration was performed using the Microsoft	Excel (2016) solver function, then rounded for convenience.  Table 3 values would	need to be redeveloped for each drug for which the parameters are being	developed.  If, for example, Albendazole were the subject, there is no expectation	that the parameter values would be the same as in Table 3.*

**MINOR REMARKS**

**1. Citations used at the end of the sentences should be before dots. For example, line 47 or at line 50.**

**T***hese have been corrected*

**2. It is a common practice to introduce the readers at the end of the introduction about the structure of the remainder of the paper (another paragraph after a paragraph ending at line 90).**

*This introduction to the structure has been added. Line 169 - 176*

**3. Line 184. Your sentence starts “ELECTRE was chosen as has an”**

*This has been changed*

**4. Line 240. Figure 5, instead of Figure 4.**

*References to figures have been corrected*

**5. Line 302. Dot is missing at the end.**

*Corrected*

**CONCLUSION**

**The idea of the manuscript is sound. It has the potential to pinpoint some relevant results as well as give a path for future applications of this methodological concept in the field. However, the manuscript is far away from publishable. It is difficult to understand it in some parts. They talk about the results in the section about the method where they use the data from the tables that are yet to be defined. References worryingly differ from the guidelines for authors. Such small problems are present throughout the manuscript. This manuscript has to be heavily restructured and revised in order to be ready for publication.**

References

[1] Yu, W. (1992). Aide multicritère à la décision dans le cadre de la problématique du tri: concepts, méthodes et applications, LAMSADE, Université Paris Dauphine, Paris (Ph.D. thesis)
[2] Fernández, E., Figueira, J. R., Navarro, J., & Roy, B. (2017). ELECTRE TRI-nB: A new multiple criteria ordinal classification method. European Journal of Operational Research, 263(1), 214-224.
[3] Almeida-Dias, J., Figueira, J. R., & Roy, B. (2010). ELECTRE Tri-C: A multiple criteria sorting method based on characteristic reference actions. European Journal of Operational Research, 204(3), 565-580.
[4] Almeida-Dias, J., Figueira, J. R., & Roy, B. (2012). A multiple criteria sorting method where each category is characterized by several reference actions: The Electre Tri-nC method. European Journal of Operational Research, 217(3), 567-579.

6. PLOS authors have the option to publish the peer review history of their article ([what does this mean?](https://journals.plos.org/plosone/s/editorial-and-peer-review-process#loc-peer-review-history)). If published, this will include your full peer review and any attached files.


If you choose “no”, your identity will remain anonymous but your review may still be made public.


**Do you want your identity to be public for this peer review?** For information about this choice, including consent withdrawal, please see our [Privacy Policy](https://www.plos.org/privacy-policy).

Reviewer #1: **Yes:** Lamia Berrah

Reviewer #2: No

[NOTE: If reviewer comments were submitted as an attachment file, they will be attached to this email and accessible via the submission site. Please log into your account, locate the manuscript record, and check for the action link "View Attachments". If this link does not appear, there are no attachment files.]

While revising your submission, please upload your figure files to the Preflight Analysis and Conversion Engine (PACE) digital diagnostic tool, <https://pacev2.apexcovantage.com/>. PACE helps ensure that figures meet PLOS requirements. To use PACE, you must first register as a user. Registration is free. Then, login and navigate to the UPLOAD tab, where you will find detailed instructions on how to use the tool. If you encounter any issues or have any questions when using PACE, please email PLOS at [figures@plos.org](mailto:figures@plos.org). Please note that Supporting Information files do not need this step.
