# Supplementary material for: Fake drugs: Using Baseline Spectral Fingerprinting and a sorting algorithm to infer quality of medications
Source: PLoS One. 2023 Apr 26;18(4):e0281416. doi: 10.1371/journal.pone.0281416 (PMC10132596; doi:10.1371/journal.pone.0281416)
Supplement: Attachment — Submitted filename: 09122021 Response to reviewer.docx [file pone.0281416.s004.docx]

**CMS response to REVIEWER #2**

Reviewer #2: The paper has improved. However, I still have significant issues with the manuscript, especially with the new parts.

1. Reading my comments you could have seen that I was talking about the ELECTRE Tri-B, and your response was that you used ELECTRE Tri-C, which is not accurate. If you have boundary profiles (Figure 2 for example is a place where you can see it), you are clearly talking about Tri-B. Please correct such mistakes.

- Line 29 & Line 92 and throughout Updated to ELECTRE TRI B

2. The whole model setting is very problematic.

2.1. Please clearly define in the manuscript what are the alternatives, and more importantly criteria.

This is an unique and unfamiliar application of a sorting algorithm, and we agree with the reviewer that the reader might not be able to recognize the alternatives and criteria. Therefore, the following clarification will be added to the paper for clarity. We clarify that the alternatives:

Line 107 – 115 and continue to clarify thoughout the manuscript

- The purpose of multicriteria decision processes are to differentiate between alternatives through some analysis of the criteria used to describe or define each alternative. The mirage of available processes allows for this differentiation to be in the form of ranking, outranking, sorting , or clustering There are innumerable potential application of these methods that range from; a simple ranking of alternatives homes for purchase by criteria of cost, size, location, etc…, to compression of digital images by clustering pixels with similar characteristics.

The specific application in this research is for sorting samples of medications (the alternatives) into categories that differentiate (in this case, sort) the alternatives into authentic and inauthentic samples. The criteria used for this are four measures of absorbance captured at four wavelengths. Generally, alternatives are sorted using criteria of different metrics (weight, length, cost, etc…), but once these values of unique metrics are normalized to some non-dimensional scale, these are in effect assessed by a common unitless metric on some scale (0 to 1, 1 to 10, etc). In our application, the data collected from the device are normalized on a common unitless scale of absorbance 0 and 2, but at four different places on the Ultraviolet (UV) light spectrum.

For this process, there are two sets of alternatives. The first set of 25 samples are used to calibrate the parameters of the ELECTRE TRI-B sorting algorithm. The second set of 50 are used to then test the calibrated sorting parameters. In both cases, the alternatives are the 25 and 50 prepared samples, and the criteria are the metrics of absorbance on four wavelengths in the UV spectrum.

2.2. Biggest confusion causes Table 3. Are the values for the indifference, preference and veto threshold same for all wavelengths? One cannot understand it from the table.

Line 312 - 317 Clarified

- Yes, while it is possible that in future applications this will not be the case, for the specific application here, the preference thresholds were common across the four wavelengths to within any reasonable understanding of significant differences, including the indifference thresholds, which was near zero and so rounded to zero. Using rounded and common units across all four wavelengths had no impact on the results of sorting. It should be noted that future applications of this process might include 6 or more wavelengths either deeper into the UV spectrum or into visible light spectrum. Under these circumstances, it is possible, if not likely, that such a simplifying assumption will not hold, and one or all criteria (measures of absorbance at specific wavelengths) might need to be unique to that wavelength. This might also be the case for the four wavelengths of this application, but for different medications. However, preliminary study that has occurred since the initiate drafting of this manuscript suggests that common uncertainty parameters will suffice.

  3. I am not familiar with the concepts of indifference and non-compatibility in ELECTRE sorting models. Please clarify and check that part.

A different paper might have used slightly different terms, but the source paper used for this application used these terms, and thus are repeated here. These terms are defined below directly from the source document based on the performance of the credibility score relative to the credibility threshold (M.E. Fontana, C.A, Cavalcante, 2011). The authors used different nomenclature than we use in the manuscript, but these concepts of indifference and incomparable are clear. As implemented: 1) indifference simply means that the algorithm does not definitively sort an alternative to a specific category, and thus is indifferent and leaves this to the decision maker, and 2) incomparable means that the sample simply cannot be sorted to any category. This is primarily because the alternative so fundamentally dissimilar to the logic of the category system that there is no meaningful placement possible. To be crude, it is like trying to sorting a table into a system designed for farm animials. Below is the authors’ of the sort document logic for preference, indifference and incomparability.

The values of *σ*(*a, b*B*h*B), *σ*(*b*B*h*B*, a*) ) and λ determine the preference situation between *a* aand *b*B*h*B:

*a) σ*(*a, b*B*h*B)≥λ and *σ*(*b*B*h*B*, a*)≥λ *→ aSb*B*h* Band *b*B*h*B*Sa → aIb*B*h*B*,* i. e., *a* is indifferent to *b*B*h*B*;*

b) *σ*(*a, b*B*h*B)≥λ and *σ*(*b*B*h*B*, a*)<λ *→ aSb*B*h* Band not*b*B*h*B*Sa → a* ≻*b*B*h*B*,* i. e., *a* is preferred to *b*B*h* B(weakly or strongly);

c) *σ*(*a, b*B*h*B)<λ and *σ*(*b*B*h*B*, a*)≥λ *→* not *aSb*B*h* Band *b*B*h*B*Sa→ b*B*h*B≻ *a,* i. e., *b*B*h* Bis preferred to *a* (weakly or strongly);

d) *σ*(*a, b*B*h*B)<λ and *σ*(*b*B*h*B*, a*)<λ *→* not *aSb*B*h* Band not *b*B*h*B*Sa→ aRb*B*h*B*,* i. e., *a* is incomparable to *b*B*h*B*.*

- 4. Please do a thorough proof reading before sending the manuscript. Page 5, line 100 you did not finish the name of the section; page 7, line 130, no dot at the end of the sentence; page 8, line 157 same thing. There are double spaces on several spots.
- Line 73 -	The Section has the correct name
- Line 130 - All sentence have periods at the end of it
- Line 157 - All sentence have periods at the end of it. You may be referring the Fig 1 legend which appears to blend with the main body of the manuscript due to the formatting required.

  Finally, although improved, manuscript still has several sloppy mistakes and I urge authors to carefully read everything, including background methodological material and correct all the issues present in the current version of the manuscript.

We take exception to the reviewers reference to “sloppy mistakes.” This is a very complex method that we describe for a non mathematical global health audience in a very specific format. We are happy to change any errors you may point out.

**CMS response to REVIEWER #3**

Reviewer #3: The paper presents an interesting testing procedure to authenticate drugs applying a sorting algorithm based on ELECTRE TRI.

The problem is clearly stated, and the main contribution of the proposal is discussed. However, there are still some concerns with the sorting procedure proposed in the BSF test. Additionally, some statements in the manuscript are vague and could be rewritten. These statements are commented below.

\# Line 82 – a multi-criteria sorting algorithm which can be used to compensate for the uncertainty when a pharmaceutical compound is analyzed “in the field” …
It is not clear that a multi-criteria sorting algorithm can be used to compensate for uncertainty. This terminology can be confusing in the decision analysis field.
As a suggestion, I would change the term uncertainty for imprecise , that is more appropriate.

Line 357, 361, ……We agree, and the paper has been update. That said we did not find that “imprecise” was a clear term either and we therefore chose to use the word “randomness”

- #Line 91 - To compensate for samples prepared in the field using less than ideal equipment (i.e randomness in concentrations) the ELECTRE TRI-C multi-criteria sorting algorithm was adapted and applied to increase the likelihood of sorting a sample to the correct authentic/inauthentic category.
  The sorting method presented is more related to the ELECTRE TRI-B, in which boundaries of the categories are defined. ELECTRE TRI-C does not depend on boundaries profiles but references examples. Please correct it in the manuscript.
  I would not use the term “increase the likelihood of sorting a sample”, as the method ELECTRE was not designed for dealing with likelihood.
- The manuscript has been updated as the reviewer suggests

\# Line 99-102 –difficult to read

- We have changed the wording

\# Line 149 – ELECTRE TRI-C is an algorithm that has specific attributes that allow for uncertainty and variability in collected data (or variability in sample preparation) that makes it ideal for the purposes of this method [17,18, 19, 20].
I disagree with this statement. Electre tri being the IDEAL method is very strong. The BSF is itself an interesting method to analyze the drugs. Besides the relevance of the studied problem, the sorting algorithm based on ELECTRE TRI does not seem to be the most appropriate one. (please consider that this comment does not invalidate the results). The point is, the study is using a sophisticated sorting technique that deals with the conflict of criteria and preferences of a decision-maker. However, the BSF problem seems to be more related to the calibration of measures by a specialist. The main point is that a similar result (dugs being considered authentic or not) could be reached by a procedure based on fuzzy logic. A Fuzzy method, including a similar calibration step, could be applied to define if a sample is authentic or not. Fuzzy systems (Zadeh, 1965) are well-known methods used to account for the knowledge of a specialist (in this case, the pharmaceutics). This is because a sample to be considered authentic must have all measures (for each wavelength of 240, 250, 260 and 270 – the criteria) under a specific value interval, but with some imprecise boundaries.
(observe that criteria weights are equal, and the credibility thresholds is 0,85)

- Line 172 - Rephrased as “convenient” rather than “ideal”. Further, we do not disagree with the reviewer, and future versions of this process will investigate using fuzzy logic as a possible approach.

Line 173 – mention ELECTRE TRI-C.

- Added as per reviewers suggestion

\# Line 181 – Presents the sorting procedure.
In this case, the author could mention that an adapted procedure is used to sort the samples, based on the combined notion of the pessimistic and optimistic procedure of ELECTRE TRI-B. The explanation of left-to-right is interesting for a non-specialist in the area.

- Reviewed by authors for clarity

  # Line - 282 Raw transmitted light absorbance data collected for 25 samples prepared in order to optimize ELECTRE TRI uncertainty parameters.
  And
  # Line 287. “This data displays three uncertainty parameters for ELECTRE TRI sorting algorithm. Values were selected by optimizing the minimize number of false positive and false negatives out of 50 samples of random substances (with 4 being factually positive)”.
  It is not clear how the parameters have been optimized. This is quite important because the sorting procedure will depend on these established values.
  In the response letter, the authors says that the solver function was applied to calibrate the categories. This should be mentioned in the text and explained in the supplement material, for instance.
- This has been updated. There seems to have been some confusion between the authors as to what was main text and what was table legend.

\# Line 350 - “There are a number of sorting algorithms that consider uncertainty in data, PROMETHEE II being one, that were also investigated.”
PROMETHEE II does not mean to sort alternatives.

- This has been corrected as PROEMTHEE Sort (PROMSORT)

\# Line 361 - “Whereas ELECTRE TRI-C fails to sort this sample to any category, other methods would require that this be sorted to the best, if not correct, category. Thus, ELECTRE –TRI-C avoids the false positives challenge to a higher degree than other methods explored.”
I cannot see how ELECTRE can deal with false positives and negatives with such a sample. I don’t think this is the best argument. The procedure is not statistical.

- This section has been reviewed and updated for clarity. However, to address the reviewers comment: the method was employed for this study specifically because it is not a statistical process. For any sorting system or sorting algorithm, there will be items that are sorted into a category based on the measured attributes. This sorting is not based on statistics, but on attributes of 1) the item/alternative, and 2) the defined parameters and boundaries of the system into which the item/alternative is being sorted. Further, there are instances wherein a sample cannot be sorted to any category simply because the attributes of the item/alternative is so fundamentally different than the system into which it is being sorted, that the system simply cannot accommodate the item. To put it crudely, it would be like sorting a chair into a system designed for sorting circus animals. ELECTRE TRI-B is convenient for use because for any sample that cannot be sorted (is incomparable to the system), we can simply assume that that specific sample/alternative/item is LQ/C (inauthentic) sample of the medication because it is so widely dissimilar to the sorting system and defined parameters.

There are some real applications of ELECTRE TRI method that could support this application. Consider Human Development Index Based on ELECTRE TRI Multicriteria Method, Social Indicators Research, 2016.
