# Supplementary material for: Fake drugs: Using Baseline Spectral Fingerprinting and a sorting algorithm to infer quality of medications
Source: PLoS One. 2023 Apr 26;18(4):e0281416. doi: 10.1371/journal.pone.0281416 (PMC10132596; doi:10.1371/journal.pone.0281416)
Supplement: Attachment — Submitted filename: Review PLOS.docx [file pone.0281416.s005.docx]

This paper presents a novel method for inferring quality of medications specifically.The current study represents an interesting subject to improve the healthcare system. However, the authors have to detail the results. Some comments have to be revised.

1-	Authors have integrated the multi-criteria tool for sorting. However, the theoretical content is missing in the paper. It would be preferable to include a section about the multi-criteria tools.

2-	The authors should present a comparative study of the multi-criteria sorting tools.

3-	There are several multi-criteria sorting algorithms that can realize the study. The authors have chosen the use of ELECTRE TRI-B. They should justify the choice of this sorting algorithm.

4-	To prove this classification proposed by the authors, it is preferable to study the sensibility analysis according to the cut-value λ.
