# Supplementary material for: Fake drugs: Using Baseline Spectral Fingerprinting and a sorting algorithm to infer quality of medications
Source: PLoS One. 2023 Apr 26;18(4):e0281416. doi: 10.1371/journal.pone.0281416 (PMC10132596; doi:10.1371/journal.pone.0281416)
Supplement: Attachment — Submitted filename: PLOS Authors response to Reviewer (092022) (1).docx [file pone.0281416.s006.docx]

Authors’ Reponses to Reviewers’ Comments 09/2022

Note:

Review comments are presented in italics

**Authors response is presented in bold**

Reviewer #2

- *I thank the authors for an attempt to clarify the reasoning. Several critical issues emerged from the response.*
  - *1. What authors refer to in Fantana and Cavalcante's article are ways to obtain pairwise relations between two alternatives. This has nothing to do with the pessimistic and optimistic rules where you actually have the major problem. Each rule defines a category with certainty, i.e., the upper and lower boundary of the sorting procedure.*
    - **There are two alternatives. There is the sample (alternative 1) and the boundary between categories (proxy alternative). And the whole thing is a pair comparison between alternatives (samples) and category boundaries. We no longer use the nomenclature of “pessimistic and optimistic.”**
  - 2. Note that you do not have this reference in the reference list in the manuscript. Generally, you have mainly missed the good references in presenting ELECTRE Tri-B method, even though you had good suggestions from reviewers.
    - **The Fantana Cavalcante’s paper was used in a previous response to the reviewer’s comments, as it was not foundational to the research or manuscript. If the reviewer is requesting a particular citation, please so state. Most likely, we would be happy to accommodate.**

- Overall, the idea behind the work is good. The idea behind the method is very solid, and it can work well. The execution is poor. I was very sympathetic toward the authors, addressing the main issues several times, but this has still not improved. In the end, you remain with the clear methodological mistake of using indifferent and not compatible samples and evaluating them as correct classification.
  - **The authors fundamentally disagree with the contention that indifferent and not compatible samples are not evaluated correctly as LQ/C. By definition samples that are not sorted to a specific category are LQ/C. We stated this previously, and have updated the paper to emphasis this point. This is literally why we selected the ELECTRE TRI process**.

Reviewer #3

- The authors have justified the proposed sorting procedure applied in the paper, based on the notions of ELCTRE-TRI-B. In this way, the authors should avoid generalizations regarding applications of ELECTRE-TRI. For instance, I would recommend changing the following sentence (line 236, pg 10): "In the general application of ELECTRE TRI-B this situation defines Sample-Y as being defined as “incomparable” to the sorting system. In other words, the sample cannot logically be sorting to any category of the sorting system as defined by the decision maker" to: "This situation defines Sample-Y as being defined as “incomparable” to the sorting system"
  - **229-248 We appreciate the reviewer’s constructive guidance, and we believe that we have updates the language to satisfy their concern**

Reviewer #4:

- This paper presents a novel method for inferring quality of medications specifically. The current study represents an interesting subject to improve the healthcare system. However, the authors have to detail the results. Some comments have to be revised.
  - 1- Authors have integrated the multi-criteria tool for sorting. However, the theoretical content is missing in the paper. It would be preferable to include a section about the multi-criteria tools.
    - **We appreciate the observation, and have included the underlying algorithm as a supplemental**
  - 2- The authors should present a comparative study of the multi-criteria sorting tools.
    - **We disagree that it is necessary to include analysis using competitor algorithms, if for no other reason than ‘which ones”? there are scores of approaches that could be used, including other MCDA oriented methods, as well as pattern recognition software (including Artificial Neural Networks). We selected the ELECTRE TRI-B method through consultation with normative experts, and used this algorithm based on their recommendations. Repeating the analysis using other methods might be informative from an abstract conceptual learning context to the MCDA community. But the purposes here is to document the results of an effective process, and as such determining if other methods are as effective or not is a distraction**
  - 3- There are several multi-criteria sorting algorithms that can realize the study. The authors have chosen the use of ELECTRE TRI-B. They should justify the choice of this sorting algorithm.
    - **See note above as to why the ELECTRE TRI-B algorithm was selected. In addition, we updated the paper to make this point clearer to the reader. But in short: it was recommended, we tried it and it worked. Future work can return to this issue as a comparative analysis of various methods (including non-MCDA methods) as an interesting follow up body of research, but this work currently stands as it is.**
  - 4- To prove this classification proposed by the authors, it is preferable to study the sensibility analysis according to the cut-value λ.
    - **We agree that this could be an interesting addition to the paper, and λ was adjusted to see impact on results. Lowering to 0.75 had no material impact on the results because anything that is not sorted to CAT-A is by definition LQ/C, and thus everything was still correctly sorted. However, by increasing to 0.87 one Authentic sample was reclassified as LQ/C. This is an interesting observation but still speaks to the benefit of using the preliminary 25 samples to train the model parameters, and that this was an effective way to define the parameter values.**
