# Supplementary material for: Fake drugs: Using Baseline Spectral Fingerprinting and a sorting algorithm to infer quality of medications
Source: PLoS One. 2023 Apr 26;18(4):e0281416. doi: 10.1371/journal.pone.0281416 (PMC10132596; doi:10.1371/journal.pone.0281416)
Supplement: Attachment — Submitted filename: Author response to reviewer 4 comments.docx [file pone.0281416.s007.docx]

*Review #4 Comment*

*The authors have not revised the fourth comment (To prove this classification proposed by the authors, it is preferable to study the sensibility analysis according to the cut-value λ.). The authors are asked to add a new section to include the sensitivity analysis study.*

Author Response:

We had included a discussion on sensitivity of the cut-value / credibility threshold in the last version, but it was located in the discussion section. This has been updated with more information and moved to the end of the proof of concept section.

Line 641-651 & 765-774
